# Validation of the Taiwan Chinese Version of the Assistive Technology Usability Questionnaire for People With Neurological Diseases for Wearable Robotic Exoskeletons: Usability Study

**DOI:** 10.2196/89556

**Published:** 2026-07-20

**Authors:** Hander Wang, Jian-Jia Huang, Yu-Cheng Pei, Huey-Wen Liang

**Affiliations:** 1 School of Medicine, Chang Gung University, Taouyuan, Taiwan; 2 Department of Physical Medicine and Rehabilitation, Chang Gung Memorial Hospital, No. 5, Fuxing St., Guishan District, Taouyuan, 33305, Taiwan, 886 3-3281200 ext 3846; 3 Master of Science Degree Program in Innovation for Smart Medicine, Chang Gung University, Taouyuan, Taiwan; 4 Department of Physical Medicine and Rehabilitation, National Taiwan University College of Medicine, Taipei City, Taiwan; 5 Department of Physical Medicine and Rehabilitation, National Taiwan University Hospital, Taipei City, Taiwan

**Keywords:** usability, rehabilitation, exoskeletons, stroke, robot-assisted gait training, questionnaires, wearable robotic exoskeletons, assistive technology, psychometrics

## Abstract

**Background:**

Assessing usability is important given the increasing use of technology in rehabilitation. While wearable robotic exoskeletons (WREs) are commonly incorporated into neurological rehabilitation in both clinical and community settings, there is a need to examine the applicability of different usability instruments in this context. The Assistive Technology Usability Questionnaire for People with Neurological Diseases (NATU Quest) was developed to evaluate the usability of assistive technology in individuals with neurological conditions. However, its reliability and validity have been established only for assistive devices such as wheelchairs and canes, restricting its generalizability.

**Objective:**

This study aimed to cross-culturally adapt a Taiwan Chinese version of the NATU Quest and to preliminarily evaluate its applicability, reliability, and validity for assessing WRE use among patients who had a stroke as an expanding application context.

**Methods:**

Cross-cultural adaptation was achieved through a structured translation from Spanish to Taiwan Chinese, followed by content validation by an expert panel of 10 raters. Following the adaptation process, psychometric testing of the NATU Quest was administered to 20 patients who had a stroke following the completion of a comprehensive course of robot-assisted gait training facilitated by a WRE. The internal consistency of the NATU Quest was evaluated using the Cronbach α coefficient. The reliability of the test-retest procedure was evaluated using the intraclass correlation coefficient (ICC). Convergent validity was ascertained by examining the correlation between the NATU Quest scores and the System Usability Scale (SUS).

**Results:**

The culturally adapted NATU Quest showed acceptable content validity, with item-level content validity index (CVI) values ranging from 0.80 to 1.00 and scale-level CVI values of 0.93 for clarity and 0.97 for relevance. Cronbach α was 0.943, indicating high internal consistency. Of the 20 participants who completed all 6 rehabilitation sessions, 13 participants completed repeat testing. Preliminary test-retest reliability was acceptable (ICC=0.823; *P*<.001; SE of measurement=0.31). NATU Quest scores were strongly correlated with SUS scores (Spearman ρ=0.818; *P*<.001), providing preliminary support for convergent validity.

**Conclusions:**

The adapted NATU Quest demonstrated promising preliminary psychometric properties when applied to WRE use among individuals who had a stroke. Further studies with larger sample sizes are warranted to examine additional psychometric properties such as construct validity and responsiveness.

## Introduction

Wearable robotic exoskeletons (WREs) have been widely used for robot-assisted gait training (RAGT) to restore walking ability in individuals with neurological or mobility impairments for many years [1]. They offer high-intensity and task-specific training and have demonstrated benefits for stroke, spinal cord injury, Parkinson disease, and cerebral palsy [2-5]. Moreover, WREs are increasingly transitioning beyond clinical and laboratory settings toward widespread adoption in homes, communities, or even industrial settings, reflecting a broader application for home-based rehabilitation and assistive devices [6-8].

While the application of WREs has become increasingly popular in rehabilitation, usability assessment for their effective adoption and clinical integration has not yet been comprehensively addressed. According to the International Organization for Standardization, usability is defined as “the effectiveness, efficiency, and satisfaction with which users achieve specified goals in specific contexts of use” [9]. It is an important feature of an assisted technology in rehabilitation related to user comfort, feedback mechanisms, difficulties in donning and doffing, compliance mismatch, and environmental conditions [10-15]. The World Health Organization also recommends that usability assessment should be the initial step in evaluating any digital health intervention [16]. However, a systematic review by Koumpouros [17] indicated that 64% of studies relied on either custom-developed or nonvalidated instruments to assess the usability of assistive robotic technologies. The lack of a comprehensive psychometric evaluation of these instruments will likely raise doubts about reliability and validity, limiting comparability between studies.

Usability evaluation of WREs requires consideration of the elements that contribute to the interaction between the device and the user. These elements include aspects such as comfort, ease of donning and doffing, individual adaptability, perceived safety, and functional support [17]. However, there is limited research on usability evaluation instruments for WREs. Up to 67% of the measures used for subjective assessment of rehabilitation and assistive robot devices are custom-made and lack validation [17]. Among the standardized measures, two are most commonly used, that is, the System Usability Scale (SUS) [18] and the Quebec User Evaluation of Satisfaction with Assistive Technology (QUEST 2.0) [19,20]. The SUS was originally developed for website usability. Despite its broad application to various products, it focused more on the software design and users’ interaction with systems [21] without addressing the physical, task-dependent, and dynamic human-device interactions that are central to rehabilitation robotics [22]. The QUEST 2.0 demonstrates a stronger alignment with our intended focus on device-related usability; however, its inclusion of service-related domains, such as delivery, repairs, and follow-up services, limits its relevance in clinical scenarios where patients who had a stroke predominantly use WREs within hospital-based rehabilitation settings [19]. Recently, an Assistive Technology Usability Questionnaire for People with Neurological Diseases (NATU Quest) was developed and addresses the paucity of assessment tools covering specific domains relating to assistive technology, such as adaptability to user needs, perceived safety, contextual fit, and user experience [23]. These domains may represent a contextually appropriate measure of immediate user-device interaction in WRE-assisted gait training; however, their feasibility remains to be examined.

The NATU Quest is a self-reported questionnaire containing 10 items selected through 2 rounds of a Delphi study based on the opinion of 69 experts. It is scored on a 6-point Likert scale, with item scores ranging from 0 to 5. The total questionnaire score is calculated by summing the scores of valid responses to items 1 to 10 and dividing by the number of valid items, resulting in a final score ranging from 0 to 5, with higher scores indicating greater usability. The initial validation study assessed the usability of conventional assistive tools (eg, wheelchairs and walking aids) in patients with neurological impairments and demonstrated good internal consistency (Cronbach α=0.895), high test-retest reliability (intraclass correlation coefficient [ICC]=0.869), and construct validity (Spearman ρ=0.756 with 8 items of the QUEST 2.0) [23]. In a recent study, Bosch-Barceló et al [24] further applied the NATU Quest on a gamified virtual reality treadmill intervention used in rehabilitation for patients with Parkinson disease, expanding its application to different technology.

Our study had 2-fold goals. First, we would adapt a Taiwan Chinese version of the questionnaire following an established guideline to ensure the cross-culture equivalence of the questionnaire. Second, we examined whether the NATU Quest can be applied in a group of patients undergoing RAGT, a representative example of wearable robotic rehabilitation technology, and will preliminarily evaluate its psychometric properties. We hypothesized that the adapted questionnaire had acceptable reliability and validity. The results could provide pilot evidence to ascertain its suitability for usability assessments of WREs in future studies.

## Methods

### Cross-Cultural Adaptation

The adaptation of the Spanish version of the 10-item NATU Quest questionnaire was conducted according to the guidelines proposed in the study by Guillemin et al [25] after we obtained the permission from the original authors.

It was first forward translated into Taiwan Chinese by 2 native Taiwan Chinese translators proficient in Spanish without medical backgrounds. The first draft was concluded by a consensus-based expert panel consisting of a rehabilitation physician, an occupational therapist, and one of the translators to review both translations and resolve discrepancies. Back translation of the first draft into Spanish was conducted by an independent native Spanish translator fluent in both Taiwan Chinese and Spanish, who was blinded to the original NATU Quest. The expert panel conducted a second review to compare the back-translated version to the original Spanish NATU Quest and resolve discrepancies to ensure semantic, idiomatic, experiential, and conceptual equivalence. During this process, the panel was assisted by one of the original authors (Maria Masbernat-Almenara) through personal communication to clarify minor ambiguities. Finally, the content validity index (CVI) of the finalized version was tested by a panel of 10 physical therapists and occupational therapists who are all familiar with the use of WREs. Each item was assessed based on clarity and relevance related to the WREs using a 4-point Likert scale [26]. The results were used to compute 2 indices: item-level content validity index (I-CVI) and scale-level content validity index (S-CVI) according to equations (1) and (2). The minimum threshold of the I-CVI and S-CVI was 0.78 and 0.8, respectively [26].


$$I\text{-}CVI=\frac{\text{Number of rating 3 or 4}}{\text{Total number of experts}}\;(1)$$

$$S\text{-}CVI=\frac{\sum I\text{-}CVI}{\text{Total number of items}}\;(2)$$

### Study Design and Participants

**Figure 1.**
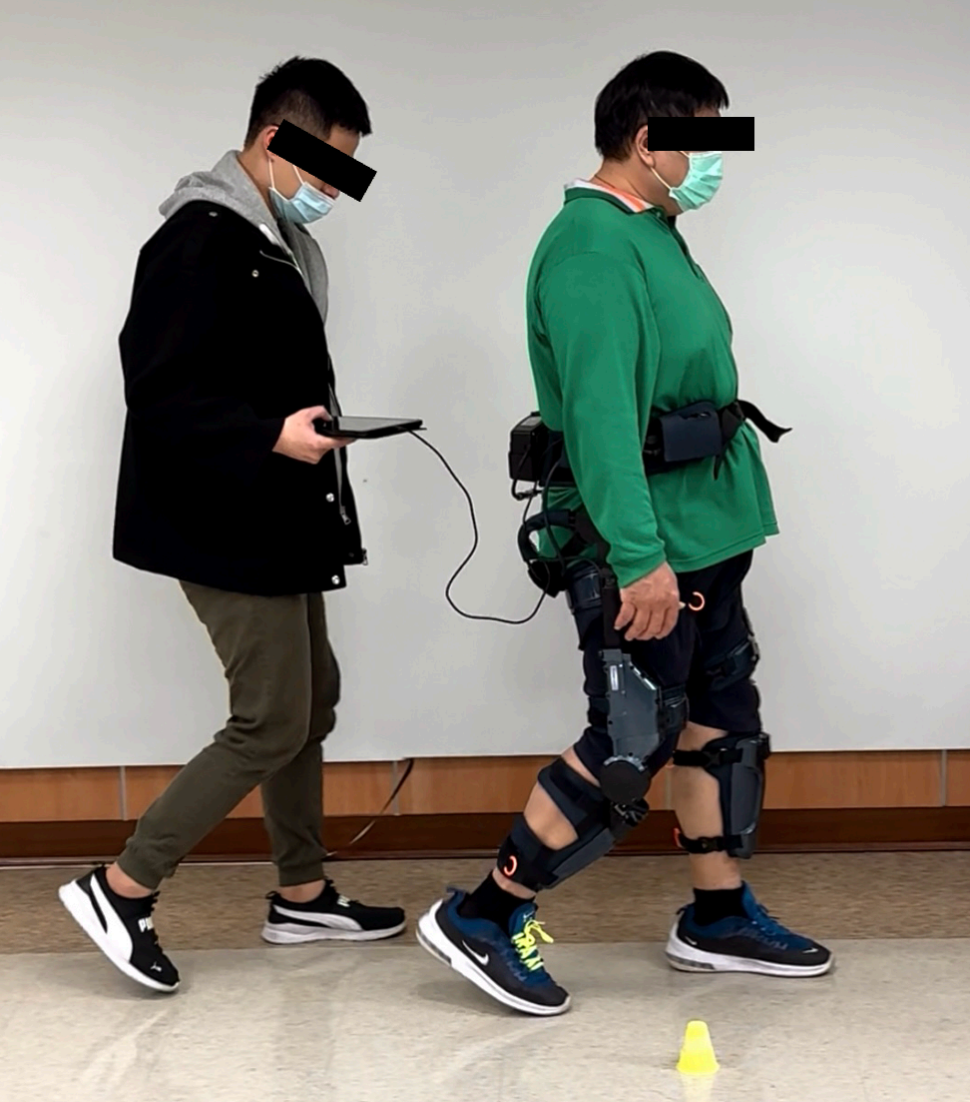
A patient who had a stroke undergoing Keeogo exoskeleton robotic gait training.

This was an extended study of a prospective and pilot case-control study aiming at investigating the therapeutic effects of RAGT using a user-initiated powered WRE in patients in the early stages of recovery after stroke [27]. All RAGT sessions used the same WRE, Keeogo Exoskeleton (Wistron Medical Technology; Figure 1). It is an electrically powered robotic device that provides knee power assistance while preserving passive hip motion [28,29]. Participants were patients who had a stroke in the early subacute stage who could maintain basic postural stability and demonstrated sufficient lower-limb motor capacity to safely engage in user-initiated robotic gait training. Individuals with substantial cognitive impairment, severe musculoskeletal or cardiopulmonary conditions, insufficient joint range or muscle tone, or dermatological or vascular problems preventing safe device use were excluded [27]. Given the limited availability of clinically eligible participants for inpatient exoskeleton training, a pragmatic pilot sample was recruited.

### Ethical Considerations

The study was approved by the institutional review board (IRB) of the Chang Gung Medical Foundation (IRB approval number 202200822B0, 2022) and adhered to the Declaration of Helsinki. Before enrollment, all participants were informed of the study purpose, procedures, and potential risks and benefits, and written informed consent was obtained from each participant. Participants’ privacy and confidentiality were protected throughout the study. All research data were deidentified and stored securely, and only authorized members of the research team had access to the study data. No financial compensation was provided to participants.

### Study Procedures

Each participant completed 6 sessions of the RAGT (40 minutes per session, 1 session per day, 2‐3 sessions per week) and was invited to answer the NATU Quest and SUS after the final session (T1). The NATU Quest was repeated within 1 week after the final session (T2) for the evaluation of test-retest reliability [30].

The SUS is a standardized 10-item questionnaire used to assess perceived usability across different technologies. Each item is rated on a 5-point Likert scale, and the total score ranges from 0 to 100, with higher scores indicating higher perceived usability [18]. The SUS has been extensively studied and has demonstrated well-established reliability and validity across diverse technologies [21]. It has also been used for usability assessment in robotic rehabilitation and assistive robotic devices, including upper- and lower-limb exoskeletons, end effectors, and sensor-based systems [31-38]. Therefore, the SUS was used to assess convergent validity and to facilitate comparisons across studies. A validated Chinese version of SUS was used for the current study [39].

### Statistical Analysis

Demographic data were analyzed using nonparametric and descriptive statistics for a small sample size. The psychometric properties of the adapted questionnaire were analyzed as follows:

1. The proportion of participants who achieved the lowest or highest possible score was computed. More than 15% of participants having the maximal or minimal score was defined as having a ceiling and floor effect, respectively.
2. The internal reliability of the scale at T1 was evaluated by calculating Cronbach α and Cronbach α if an item was deleted. Values of Cronbach α between 0.70 and 0.80 were considered good internal consistency, whereas values above 0.80 were considered very good [40]. Item-total statistics were computed to evaluate the corrected item-total correlation for each item, with values ≥0.3 considered acceptable and values ≥0.5 indicating strong homogeneity [41].
3. Test-retest reliability was evaluated with an ICC and their 95% CIs for scores obtained at T1 and T2. The ICC estimates were based on a single rating, absolute-agreement, and 2-way mixed-effects model, and interpreted as poor (*<*0.40), good (0.40‐0.75), and excellent (*>*0.75) [42].
4. The SE of measurement (SEM) was calculated to reflect the variability in the scores of individual items in the questionnaire using the following formula, where SD_baseline_ is the SD of the scores from the first measurement (T1) and ICC is the test-retest reliability between the T1 and the second measurement (T2):

$SEM\text{=}{SD}_{baseline}\times \sqrt{1\text{-}ICC}$

  Subsequently, the minimal detectable change (MDC) was calculated to determine the minimum change in score that can be interpreted as a real difference, beyond measurement error, at a 95% confidence level with a *z* score of 1.96.

$MDC\text{=}1.96\times SEM\times \sqrt{2}$

  MDC was also expressed as a percentage of a parameter’s grand mean (MDC%) by dividing the MDC by the mean scores of the NATU Quest at T1. An MDC% of *<*30% is considered acceptable [43].

1. Convergent validity was tested by computing the Spearman rank correlation coefficient (ρ) between the NATU Quest and SUS scores, and the size of the correlation coefficient was interpreted as being strong (>0.75), moderate (approximately 0.50-0.75), fair (approximately 0.25-0.50), and no relationship (approximately 0.00-0.25) [44].

All statistical analyses were performed using SPSS software (version 26.0; IBM Corp). The level of statistical significance was defined as *P<.05*.

## Results

The NATU Quest was translated from the original Spanish version, and there was generally no dispute to form the consensus, except for the Q5 (“Me siento seguro usando/llevando...//...es seguro en su utilización”). Two versions of translation: “I feel that I can use the exoskeleton robot very safely” and “I feel that the exoskeleton robot is safe to use” were consolidated into “I feel safe when wearing the exoskeleton robot.” The finalized Taiwan Chinese version of the NATU Quest was submitted to the original author of the NATU Quest for a final approval.

The I-CVI for all the items regarding the clarity and relevance of the adapted version exceeded the threshold of 0.78, while the S-CVI, calculated using the averaging method, was 0.93 for clarity and 0.97 for relevance. Therefore, the adapted version was used for further testing without modifications.

Twenty participants were enrolled in the validation study, most of whom were first-time survivors of ischemic stroke in the early subacute phase following a stroke (Table 1). They completed the 6 RAGT within an average of 20.3 (SD 4.5) days, and no adverse events were reported during the experimental process.

**Table 1.** Demographic characteristics of the participants (N=20).

Variables	Results
Age (years), mean (SD)	55.8 (11.4)
Sex, male, n (%)	15 (75)
Educational level, n (%)	
Middle school and below	4 (20)
High school or vocational school	8 (40)
College or above	8 (40)
Stroke-related characteristics	
Affected side, n (%)	
Left	12 (60)
Right	8 (40)
Stroke type, n (%)	
Ischemic	14 (70)
Hemorrhagic	6 (30)
First-time stroke, n (%)	18 (90)
Stroke duration in days, median (IQR)	37 (22‐62)

The median scores of each item of the NATU Quest ranged from 4 to 5, and the overall scores were 4.4 and 4.1 on the two occasions (Table 2). No participants reported floor scores on the NATU Quest, whereas 2 (10%) participants reported ceiling scores in T1, below the 15% criterion for a ceiling effect. Meanwhile, none reported a ceiling or floor score with the SUS.

**Table 2.** Descriptive data and internal consistency measures of the Assistive Technology Usability Questionnaire for People with Neurological Diseases (NATU Quest) and System Usability Scale.

Components	Score, range	Values, median (IQR)	Item-total correlation	Cronbach α (if an item was deleted)
NATU Quest				
Q1. Effectiveness	3-5	5 (4-5)	0.791	0.937
Q2. Comfort	2-5	4 (4-5)	0.785	0.936
Q3. Adaptability or adjustability	2-5	4 (4-5)	0.768	0.936
Q4. Donning or doffing	1-5	4 (4-5)	0.736	0.940
Q5. Perceived safety	3-5	4.5 (4-5)	0.680	0.940
Q6. Functionality	2-5	4 (4-5)	0.804	0.935
Q7. Ergonomics or fit to body	3-5	4 (3.75‐5)	0.829	0.934
Q8. Ease of use	2-5	4.5 (3.75‐5)	0.868	0.932
Q9. Ease of learning	2-5	4 (4-5)	0.685	0.940
Q10. Satisfaction (overall)	3-5	5 (4-5)	0.780	0.936
Total scores				
T1	2.3-5	4.4 (3.9‐4.7)	—^a^	—
T2	3.2-4.9	4.1 (3.4‐4.6)	—	—
System Usability Scale				
T1	45-82.5	66.3 (60.6‐75.0)	—	—

a Not applicable.

The Cronbach α of the NATU Quest at T1 was 0.943, and the Cronbach α if item deleted was between 0.932 and 0.940 (Table 2), lower than the Cronbach α of the overall scale. The item-total correlations were all above 0.5, with the highest for Q8 (ease of use: 0.868) and the lowest for Q9 (ease of learning: 0.685). This finding suggested that all items contributed to the internal consistency of the scale in this sample.

Thirteen participants completed the NATU Quest on 2 occasions, with a mean interval of 3.5 (SD 1.5) days, ranging from 2 to 6 days. There were no significant differences in demographic characteristics between participants who completed the survey at T2 and those who did not, including age (*P*=.70), sex (*P*=.61), stroke duration (*P*=.23), modified Rankin Scale (*P*=.59), and SUS scores (*P*=.88) by the Mann-Whitney *U* test. The ICC was 0.823 (95% CI 0.533‐0.942; *P*<.001), indicating an excellent test-retest reliability. The SEM, MDC, and MDC% were 0.31, 0.86, and 20.1%, respectively. The collective results supported good reliability of the adapted NATU Quest.

The Spearman ρ coefficient between the NATU Quest and SUS scores was 0.818 (*P*<.001) in all 20 participants, indicating a strong correlation.

## Discussion

### Principal Findings

We adapted a Taiwan Chinese version of the NATU Quest to facilitate future cross-cultural comparisons in the usability assessment of assistive technology. We also conducted a preliminary evaluation of its psychometric properties in patients who had a stroke using WREs for RAGT, a rapidly evolving field. The results demonstrated acceptable CVI scores, test-retest reliability, and convergent validity when referenced with the SUS. By following the guidelines for the adaptation process, we ensured the contextual clarity of the adapted questionnaire. The reliability and convergent validity of the adapted NATU Quest support its potential use in the usability assessment of WREs. However, the findings should be interpreted with caution because of the modest sample size.

The 10 items of the NATU Quest were derived using the Delphi method, and the original validation study included wheelchairs, walking aids, splints, and treadmills, without specific focus on WREs. Similar to the SUS and QUEST 2.0, it is not a device-specific instrument. However, these validated instruments offer the advantages of requiring fewer resources and enabling comparability across studies [45]. Our results showed a high CVI from 10 therapists who were familiar with this WRE and confirmed the face validity. They generally align with the essential features of the medical device as emphasized by the guidelines established by the International Electrotechnical Commission and the US Food and Drug Administration guidelines [46,47]. However, caution should be exercised, as the use of a generic tool may fail to capture certain risks and usability dimensions specific to WREs [48]. WREs are available in many forms and multiple purposes, such as rehabilitation, assistive mobility, industrial and military use, sports and function, and personal mobility [49]. The WRE we tested, Keeogo, has been used in stroke, multiple sclerosis, and older adults with morbidities [27,50,51]. Its intended use includes not just for rehabilitation but also personal use. We did not attempt to add device-specific items during the adaptation process, as this could compromise the construct validity of the original questionnaire. Therefore, future users should be aware of the potentially limited coverage of certain device-specific concerns, including but not limited to biomechanical misalignment, falls, and soft-tissue pressure–related injuries.

Several psychometric properties tested in this study aligned closely with those reported for the original NATU Quest [23]. The median scores of the participants for Keeogo are between 4.1 and 4.4, around 82% to 88% of the highest scores, implying a generally positive perception of the device. Test-retest reliability (ICC=0.823) also paralleled the previously reported excellent temporal stability [23]. However, up to 10% of the participants reported ceiling scores at T1, and 2 items had median scores of 5, raising concerns regarding ceiling effects and restricted score variability in this specific clinical context. In addition, the internal consistency (Cronbach *α*=0.943) was higher than that reported in a previous study involving various devices and patients with diverse diagnoses [23]. Although the Cronbach α values if an item was deleted were mostly below 0.94, potential item overlap or redundancy should be examined in future studies with a larger sample size.

A strong correlation between the NATU Quest and SUS scores (Spearman ρ=0.818) supported convergent validity. Both the SUS and QUEST 2.0 have been used to assess the usability of rehabilitation robots [52,53], and QUEST 2.0 demonstrated greater overlap with the components evaluated by the NATU Quest than the SUS (Table 3). However, we selected the SUS over the QUEST 2.0 to test convergent validity because up to 4 items in the QUEST 2.0 were service-related, including service delivery, repairs, and follow-up services, and were therefore not applicable to the present hospital-based inpatient RAGT context. In contrast, the SUS is widely used and may facilitate comparisons across studies. Nonetheless, the convergent validity with respect to the SUS should be interpreted cautiously because of the limited overlap between questionnaire items. Future studies assessing the convergent validity of usability questionnaires for WRE may benefit from considering instruments in addition to, or instead of, the SUS.

**Table 3.** Components of different usability questionnaires.

Components	NATU Quest^a^	SUS^b^	QUEST 2.0^c^
Effectiveness	✓	✓	✓
Comfort	✓		✓
Adaptability or adjustability	✓		✓
Donning or doffing	✓		✓
Perceived safety	✓		✓
Functionality	✓		✓
Ergonomics or fit to body	✓		
Ease of use	✓	✓	✓
Ease of learning	✓	✓	
Satisfaction (overall)	✓	✓	✓
Device size or weight			✓
Durability			✓
Service or repair			✓

a NATU Quest: Assistive Technology Usability Questionnaire for People with Neurological Diseases.

b SUS: System Usability Scale.

c QUEST 2.0: Quebec User Evaluation of Satisfaction with Assistive Technology.

### Limitations

This study has several limitations. First, the small sample size and the absence of longitudinal follow-up prevented the evaluation of essential psychometric properties, including construct validity, discriminant validity, and responsiveness. These properties should be examined in future studies before the adapted NATU Quest can be more broadly recommended for WRE usability assessment. Second, the inclusion of only patients who had a stroke receiving a single type of powered lower-limb WRE limited the generalizability of the findings, as WREs are designed for diverse patient populations and clinical applications. Future studies should evaluate the applicability of the NATU Quest across different neurological and musculoskeletal conditions and a broader range of WRE systems. Finally, the NATU Quest was originally developed as a general assistive technology usability instrument and may not fully capture WRE-specific concerns, such as biomechanical alignment, fall-related risks, or soft-tissue pressure–related issues. Given the wide variety of WREs currently available on the market, developing device-specific usability instruments for each system would be time-consuming. Therefore, the NATU Quest may provide a practical general usability measure, although complementary WRE-specific safety and biomechanical assessment items may still be required.

### Conclusions

The present study adapted a Taiwan Chinese version of the NATU Quest and preliminarily evaluated its psychometric properties in patients who had a stroke undergoing RAGT with 1 powered lower-limb WRE. The findings provided preliminary evidence supporting its content validity, internal consistency, test-retest reliability, and convergent validity within this specific clinical context. Given that no device-specific instrument is currently available for assessing WRE usability, the NATU Quest may provide a useful general usability measure for patients who had a stroke receiving RAGT with a powered lower-limb WRE. However, given the safety-critical and biomechanical nature of user-device interactions in WREs, the NATU Quest may not fully capture these aspects of usability. Further studies are needed to evaluate its comprehensive psychometric properties, including construct validity, discriminant validity, and responsiveness, before the adapted NATU Quest can be broadly recommended for usability assessment of WREs across different devices, patient populations, and clinical settings.

## Supplementary material

10.2196/89556 Multimedia Appendix 1 The Taiwan Chinese version of the Assistive Technology Usability Questionnaire for People with Neurological Diseases.

## Abbreviations

CVI: content validity index
I-CVI: item-level content validity index
ICC: intraclass correlation coefficient
IRB: institutional review board
MDC: minimal detectable change
NATU Quest: Assistive Technology Usability Questionnaire for People with Neurological Diseases
QUEST 2.0: Quebec User Evaluation of Satisfaction with Assistive Technology
RAGT: robot-assisted gait training
S-CVI: scale-level content validity index
SEM: SE of measurement
SUS: System Usability Scale
WRE: wearable robotic exoskeleton
