# Supplementary material for: Validation of the Taiwan Chinese Version of the Assistive Technology Usability Questionnaire for People With Neurological Diseases for Wearable Robotic Exoskeletons: Usability Study
Source: JMIR Hum Factors. 2026 Jul 20;13:e89556. doi: 10.2196/89556 (PMC13384474; doi:10.2196/89556)
Supplement: Multimedia Appendix 1 [file humanfactors-v13-e89556-s001.docx]

Multimedia Appendix 1. The Taiwan Chinese Version of the NATU Quest.

外骨骼機器人易用性問卷

這是針對穿戴式外骨骼機器人的易用性評估

請使用 0 到 5 的評分回答問題：5 代表完全同意，0 代表完全不同意

|  | **非常**  **不同意** |  |  |  |  | **非常**  **同意** |
| --- | --- | --- | --- | --- | --- | --- |
|  | 0 | 1 | 2 | 3 | 4 | 5 |
|  | 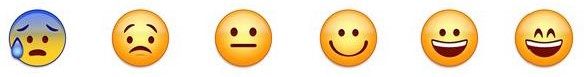 | | | | | |
| 1.我覺得外骨骼機器人有助  於改善我的功能獨立性 | 0**□** | 1**□** | 2**□** | 3**□** | 4**□** | 5**□** |
| 2.我穿戴外骨骼機器人時覺  得舒適 | 0**□** | 1**□** | 2**□** | 3**□** | 4**□** | 5**□** |
| 3.外骨骼機器人適合我的體  型及需求 | 0**□** | 1**□** | 2**□** | 3**□** | 4**□** | 5**□** |
| 4.可以快速及容易地穿上/  脫下外骨骼機器人 | 0**□** | 1**□** | 2**□** | 3**□** | 4**□** | 5**□** |
| 5.我穿戴外骨骼機器人時覺  得安全 | 0**□** | 1**□** | 2**□** | 3**□** | 4**□** | 5**□** |
| 6.外骨骼機器人讓我可以進  行我原本無法做到的行動/動作 | 0**□** | 1**□** | 2**□** | 3**□** | 4**□** | 5**□** |
| 7.外骨骼機器人可以依據我  日常生活所需做調整 | 0**□** | 1**□** | 2**□** | 3**□** | 4**□** | 5**□** |
| 8.整體而言，外骨骼機器人  容易使用 | 0**□** | 1**□** | 2**□** | 3**□** | 4**□** | 5**□** |
| 9.外骨骼機器人的使用資訊與說明，讓人容易瞭解及  記住 | 0**□** | 1**□** | 2**□** | 3**□** | 4**□** | 5**□** |
| 10.整體而言，我對外骨骼  機器人感到滿意 | 0**□** | 1**□** | 2**□** | 3**□** | 4**□** | 5**□** |

*Note.* The original developer permitted the adaptation of the NATU Quest from Spanish into Taiwan Chinese.

Ref: Masbernat-Almenara M, Rubi-Carnacea F, Opisso E, et al. Developing an assistive technology usability questionnaire for people with neurological diseases. PLoS One. 2023 Jan 31;18(1):e0281197. doi: 10.1371/journal.pone.0281197.
